# Downregulation of miR-181c-5p in Alzheimer’s disease weakens the response of microglia to Aβ phagocytosis

**DOI:** 10.1038/s41598-024-62347-x

**Published:** 2024-05-20

**Authors:** Rongjie Li, Shanshan Yao, Feijie Wei, Meixiang Chen, Yuanli Zhong, Chun Zou, Liechun Chen, Lichun Wei, Chunxia Yang, Xiyuan Zhang, Ying Liu

**Affiliations:** 1 Department of Geriatrics, The Fifth Affiliated Hospital of Guangxi Medical University, No.89 Qixing Road, Nanning, 530021 China; 2 Department of Geriatrics, The First People’s Hospital of Nanning, Nanning, China; 3 Department of Neurology, The First People’s Hospital of Nanning, Nanning, China; 4 Department of Neurology, The Second Affiliated Hospital of Guangxi Medical University, Nanning, China

**Keywords:** Alzheimer’s disease, miR-181c-5p, Apoptosis, Neurotoxicity, Aβ, Neuroscience, Biomarkers, Neurology

## Abstract

Alzheimer’s disease (AD) is an age-associated neurodegenerative disease. Recently, studies have demonstrated the potential involvement of microRNA-181c-5p (miR-181c-5p) in AD. However, the mechanism through which miR-181c-5p is responsible for the onset and progression of this disease remains unclear, and our study aimed to explore this problem. Differential expression analysis of the AD dataset was performed to identify dysregulated genes. Based on hypergeometric analysis, AD differential the upstream regulation genes miR-181c-5p was found. We constructed a model where SH-SY5Y and BV2 cells were exposed to Aβ1-42 to simulate AD. Levels of tumor necrosis factor-alpha, interleukin-6, and IL-1β were determined using enzyme-linked immunosorbent assay or reverse transcription quantitative polymerase chain reaction. Phosphorylation levels of p-P38 and P38 were detected by Western blot. The level of apoptosis in BV2 cells under Aβ1-42 stress was exacerbated by miR-181c-5p mimic. Downregulated miR-181c-5p impaired the phagocytosis and degradation of Aβ by BV2 cells. The release of proinflammatory cytokines in BV2 cells with Aβ1-42 stress was alleviated by miR-181c-5p upregulation. Additionally, miR-181c-5p downregulation alleviated the phosphorylation of P38 in Aβ1-42-induced SH-SY5Y cells. In conclusion, miR-181c-5p improves the phagocytosis of Aβ by microglial cells in AD patients, thereby reducing neuroinflammation.

## Introduction

Alzheimer’s disease (AD) is an irreversible and progressive neurodegenerative disease, which is the main cause of dementia in the elderly, accounting for 60–80% of dementia cases^1^. Its main pathological features include the formation of extracellular amyloid-β (Aβ) plaques and intracellular neurofibrillary tangles in the patient’s brain^2^ Additionally, AD is associated with loss of neurons and synaptic function, mitochondrial damage, inflammation, and excessive phosphorylation of tau proteins^3–5^. Symptoms of AD typically appear after the age of 60, and the incidence rate exponentially increases with age: 5.3% of individuals aged 65–74 have AD, 13.8% in the 75–84 age group, and 34.6% in those over 85^6^. With the increasing prevalence of AD, the economic burden and losses to families deserve attention^7^. Currently, there are no effective treatments to alleviate AD^8–10^. The urgent need for new therapeutic and preventive measures for AD has led to an in-depth exploration of the mechanisms related to extracellular Aβ plaque accumulation and the occurrence of neuroinflammation in this study.

In the early stages of AD, microglia are dedicated to rebuilding efficiency and preventing further degeneration, but they mainly fail in the later stages of the disease. This could be caused by various reasons, such as prolonged exposure to inducible inflammatory cytokines and inappropriate accumulation of Aβ peptides^11^. The pro-inflammatory activation of microglia is a hallmark of AD, a process that involves a shift from oxidative phosphorylation to glycolysis^12^. p38 is a mitogen-activated protein kinase (MAPK), that responds primarily to stress stimuli^13^. The role of p38 MAPK in the brain is crucial, as the imbalance between pro-apoptotic and anti-apoptotic pathways can lead to unwanted phenotypes like microglial activation, neuroinflammation, oxidative stress, and cell apoptosis, all involving established mechanisms such as p38 MAPK. This may provide a therapeutic window for correcting abnormal neuronal dynamics in the brain^14^. Therefore, this study further investigates the impact of p38 MAPK in microglia on AD.

MicroRNA (miRNA) is a short, non-coding single-stranded RNA responsible for influencing important processes in normal bodily functions^15,16^. Previous studies have shown that miR-181c, by binding with tissue proteinase reaction-mediating protein-2 in a mouse model, leads to the pathogenesis of AD^17^. In another study, it was found that the binding of miR-181c-5p promotes the over-phosphorylation of tau in AD through the long non-coding RNA 507 (LINC00507) in the hippocampus and cerebral cortex of AD mouse models and Aβ42-induced SH-SY5Y cell models^18^. However, these studies only suggest that miR-181c-5p has a certain impact on AD and do not clearly specify the mechanism through which miR-181c-5p affects the progression of AD. Therefore, this research further investigates how miR-181c-5p influences the development of AD in microglial cells.

## Materials and methods

### Data resources and preprocessing

AD-related data was obtained from the Gene Expression Omnibus (GEO) database (https://www.ncbi.nlm.nih.gov/geo/)^19^. From this, tissue samples of the whole lateral ventricle choroid plexus were obtained from the GSE110226 dataset, including 6 healthy control subjects and 7 late-stage AD subjects. For data standardization and preprocessing, we used the limma^20^ software package.

### Differential gene expression analysis

To explore dysregulated genes in AD, we performed mRNA differential expression analysis using the limma software package^20^.

### Construction of protein–protein interaction (PPI) network

By constructing a PPI network^21^, differential expression genes were integrated into the PPI data. Network clusters were identified in these networks to improve protein function prediction. The significant clusters were introduced by Cluster-ONE application of Cytoscape software 3.4.0. The genes are retrieved from STRING date base and analyzed by Cytoscape software.

### Identification of upstream regulators

In this study, we explored upstream regulators related to AD genes using RNAInter database. Perform hypergeometric testing on all differentially expressed genes, and the upstream regulation miR-181c-5p was found.

### Preparation of amyloid β-peptide 1–42 (Aβ1-42)

Aβ monomer (Sigma-Aldrich, St Louis, MO, USA) was transferred from a − 80 °C refrigerator to a disinfected table under a sterile condition. An appropriate amount of dimethyl sulfoxide (DMSO; Sigma-Aldrich) was added into an Eppendorf (EP) tube containing Aβ monomer and thoroughly mixed until completely dissolved. The samples were then incubated with culture medium at 4 °C overnight and centrifuged at 14,000 rpm for 10 min at 4 °C. The supernatant was taken as Aβ1-42 oligomer.

### Cell culture and administration

SH-SY5Y neuronal cells and microglial BV2 cells (Shanghai Cell Bank, Chinese Academy of Sciences, Shanghai, China) were cultivated in Dulbecco’s Modified Eagle Medium (DMEM; Invitrogen, Carlsbad, CA, USA) composed of 15% fetal bovine serum (FBS) and 1% penicillin/streptomycin at 37 °C, 5% CO_2_. Aβ1-42 (0, 1, 2, 5, 10, and 20 μM) was utilized for inducing SH-SY5Y and BV2 cells for 6 h. The appropriate dose of Aβ1-42 was then determined. miR-181c-5p mimic or inhibitor (Genepharma, Shanghai, China) was transfected into the above cells to up- or downregulate its level. After 2 h, 5 μM Aβ1-42 was added and cultivated for 6 h.

### Reverse-transcription quantitative polymerase chain reaction (RT-qPCR)

Total RNA was extracted using TRIZOL reagent (Takara, Shiga, Japan) following the manufacturer’s instructions, and the content of extracted RNA was assessed. Prime Script RT Reagent Kit (Takara) was used to generate complementary DNA (cDNA). Amplification was achieved utilizing SYBR Premix Ex Taq (Takara) in line with the manufacturer’s standard protocols. The experimental data were computed using the 2^−∆∆CT^ approach and normalized with U6 or glyceraldehyde 3-phosphate dehydrogenase (GAPDH). The primer sequences were as follows: miR-181c-5p, 5’-GTCGTATCCAGTGCAGGGTCCGAGGTGCACTGGATACGACACTCACCG-3’ (forward), 5’-TGCGGAACATTCAACCTGTCGG-3’ (reverse); U6, 5’-GTCGTATCCAGTGCAGGGTCCGAGGTATCGCACTGGATACGACAAAATATGGAAC-3’ (forward), 5’-TGCGGGTGCTCGCTTCGGCAGC-3’ (reverse); MAPK1, 5‘-TACACCAACCTCTCGTACATCG-3’ (forward), 5’-CATGTCTGAAGCGCAGTAAGATT-3’ (reverse); tumor necrosis factor-α (TNF-α), 5’-CAGGCGGTGCCTATGTCTC-3’ (forward), 5’-CGATCACCCCGAAGTTCAGTAG-3’ (reverse); interleukin (IL)-6, 5’-CTGCAAGAGACTTCCATCCAG-3’ (forward), 5’-AGTGGTATAGACAGGTCTGTTGG-3’ (reverse); and IL-1β, 5’-GAAATGCCACCTTTTGACAGTG-3’ (forward), 5’-TGGATGCTCTCATCAGGACAG-3’ (reverse).

### Cell counting kit-8 (CCK-8) assay

A CCK-8 assay (Dojindo Laboratories, Kumamoto, Japan) was used to detect the survival of SH-SY5Y and BV2 cells. Briefly, 1000 cells with complete medium were cultivated in a 96-well plate, after which a mixture of 10 μL CCK-8 reagent with 90 μL DMEM was used to generate the working solution. Working solution (100 μL) was then added to each well and incubated for 1.5 h. The absorbance at 450 nm was measured with a microplate reader. Three replicate wells were set in each group, with three repetitions.

### Annexin-V/propidium iodide (PI) staining

In accordance with the instructions of the manufacturer (BD Biosciences, Franklin Lakes, NJ, USA), BV2 cells were gathered and washed with ice-cold phosphate-buffered saline (PBS) twice. Next, the cells were resuspended in binding buffer with 1 × 10^6^ cells/mL in a 6-well microplate for 24 h. The cells were then incubated with 5 µL Annexin V-FITC and 5 µL PI for 15 min away from light at 37 °C. A flow cytometer (BD) was used to measure the level of apoptosis, and the data were processed using FlowJo software 10.8.1 (BD).

### Fluorescent Aβ phagocytosis flow cytometry

BV2 cells were exposed to 2 μM fluorescent Aβ (Abcam, Cambridge, MA, USA) for 2 h at 37 °C. The phagocytic uptake of Aβ was displayed as the proportion of microglia positive for Aβ fluorescence, which was measured using a flow cytometer (BD).

### Enzyme-linked immunosorbent assay (ELISA)

TNF-α, IL-6, an IL-8 in the supernatant of BV2 cells were determined by ELISA in accordance with the instructions of the corresponding ELISA kits (Nanjing Jiancheng Institute of Biological Engineering, Nanjing, China). Different concentrations of standard and 100 μL of sample were successively added to the corresponding wells of a microplate. The microplate was coated with film and incubated at 37 °C for 1 h. After discarding the liquid from the wells of the microplate, it was washed fully 3 times with washing liquid and blotted with filter paper. Coloring agent A (50 μL) was added to each well, after which 50 μL coloring agent B was added to the wells. The mixture was shaken and mixed, and the color was developed at 37 °C for 10 min in the dark. To stop the reaction, 50 μL termination solution was added to each well. Ten minutes after adding the stop solution, the blank well was adjusted to zero, and the optical density (OD) value of each well was measured at 450 nm. The levels of TNF-α, IL-6, an IL-8 were determined through standard curves.

### Western blot

Cells were lysed with radioimmunoprecipitation assay (RIPA) buffer (Beyotime Biotechnology, Shanghai, China) plus 1% protease inhibitor for half an hour on ice. Extracted protein was subjected to sodium dodecyl sulfate polyacrylamide gel electrophoresis and then transferred onto polyvinylidene difluoride (PVDF) membranes (Millipore, Boston, Massachusetts, USA). After blocking, incubation with primary antibody of phosphorylated (p)-P38 (1:500; ab178867; Abcam), P38 (1:2000; ab170099), or β-actin (1:5000; ab179467) was implemented, followed by goat anti-rabbit secondary antibody (1:5000; ab7090). Protein was developed via Pierce ECL Western blot substrate (Sigma-Aldrich).

### Statistical analysis

All data are presented as mean ± standard deviation. In this study, it is necessary to examine whether the multiple levels of a certain factor have an effect on a certain quantitative data, so over three groups of data were compared using one-way analysis of variance with Turkey’s post hoc test,the single-factor analysis follows a normal distribution. All statistical analyses and charts were achieved using GraphPad Prism 8.0.1. In addition, different numbers of “*”represent p-values, **, *p* < 0.01; ****, *p* < 0.0001. *p* < 0.01 indicated statistical significance.

### Ethics approval and consent to participate

The authors are accountable for all aspects of the work, including ensuring that any questions related to the accuracy or integrity of any part of the work have been appropriately investigated and resolved.

## Results

### Identification of miR-181c-5p in the AD dataset

**Figure 1:**
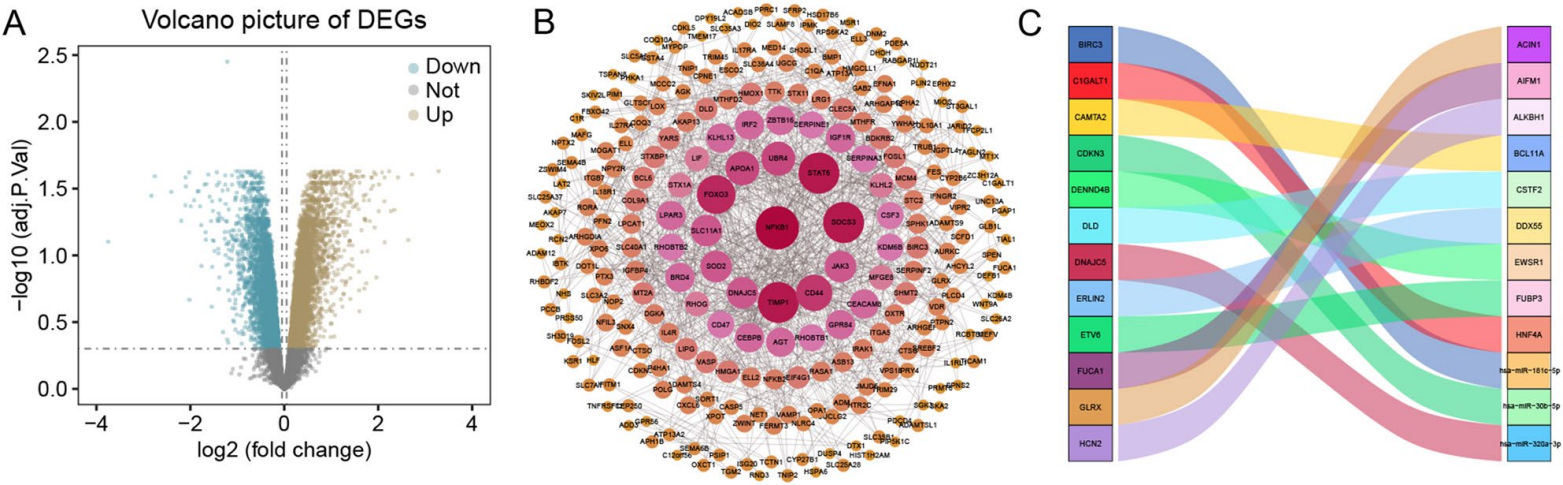
Identification of miR-181c-5p in the AD dataset (**A**) The volcano plot illustrates the distribution of AD genes and differentially expressed genes, with downregulated genes on the left and upregulated genes on the right. (**B**) The protein–protein interaction network diagram demonstrates the functional interactions among AD-related genes. (**C**) The Sankey diagram presents some of the genes obtained from hypergeometric analysis.

Through differential gene expression analysis, we identified genes related to AD and determined the differential expression patterns of differentially expressed genes. This analysis revealed 340 upregulated genes and 198 downregulated genes **(**Fig. 1A). Subsequently, to predict protein network functionality, we constructed a PPI network for these differentially expressed genes and calculated the degree values. All the key genes were organized into a cluster, which included 255 nodes (Fig. 1B). Finally, we identified the upstream regulatory genes of module genes by conducting hypergeometric analysis on differentially expressed genes, and ultimately obtained the upstream regulatory gene miR-181c-5p and visualized it (Fig. 1C).

### miR-181c-5p presents the downregulation in Aβ1-42-induced SH-SY5Y cell models

**Figure 2:**
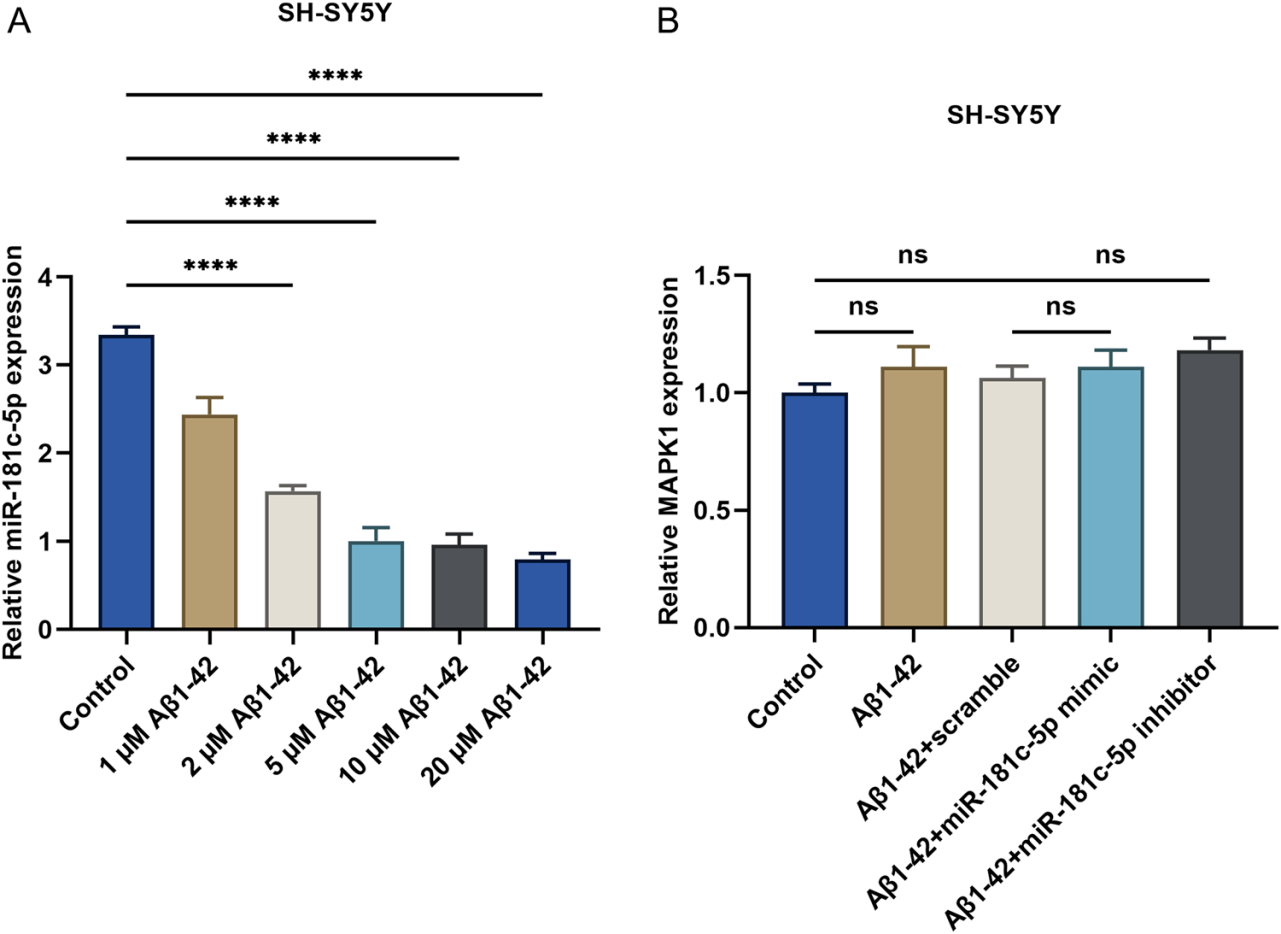
miR-181c-5p presents the downregulation in Aβ1-42-induced SH-SY5Y cells. (**A**) Measurement of miR-181c-5p level in SH-SY5Y cells with exposure to diverse doses of Aβ1-42 by use of RT-qPCR. (**B**) Measurement of MAPK1 level in SH-SY5Y cells with controls, 5 μM Aβ1-42 or/and scramble sequence, miR-181c-5p mimic, or inhibitor transfection. ****, *p* < 0.0001; ns, *p* > 0.05. miR-181c-5p, microRNA-181c-5p; Aβ, amyloid β; MAPK, mitogen-activated protein kinase; RT-qPCR, reverse transcription quantitative polymerase chain reaction.

Aβ1-42-induced neurotoxicity is an underlying mechanism of the initiation and progression of AD^22^. We measured the expression of miR-181c-5p in Aβ1-42-induced SH-SY5Y cell models using RT-qPCR. Compared with controls, miR-181c-5p level exhibited significant reduction in SH-SY5Y cells with Aβ1-42 exposure (Fig. 2A). As the dose increased, miR-181c-5p level displayed gradual downregulation. This was indicative of the involvement of miR-181c-5p in the initiation and progression of AD. For subsequent experiments, 5 μM Aβ1-42 was used. To investigate the influence of miR-181c-5p on AD, the transfection of miR-181c-5p mimic and inhibitor into SH-SY5Y cells was carried out. While mitogen-activated protein kinase (MAPK) plays an essential role in AD pathophysiology^23^, no notable influence of altered miR-181c-5p on MAPK1 level was found in Aβ1-42-exposed SH-SY5Y cells (Fig. 2B).

### The supernatant of BV2 microglial cells with miR-181c-5p downregulation improves the survival of BV2 cells under Aβ1-42 stress

**Figure 3:**
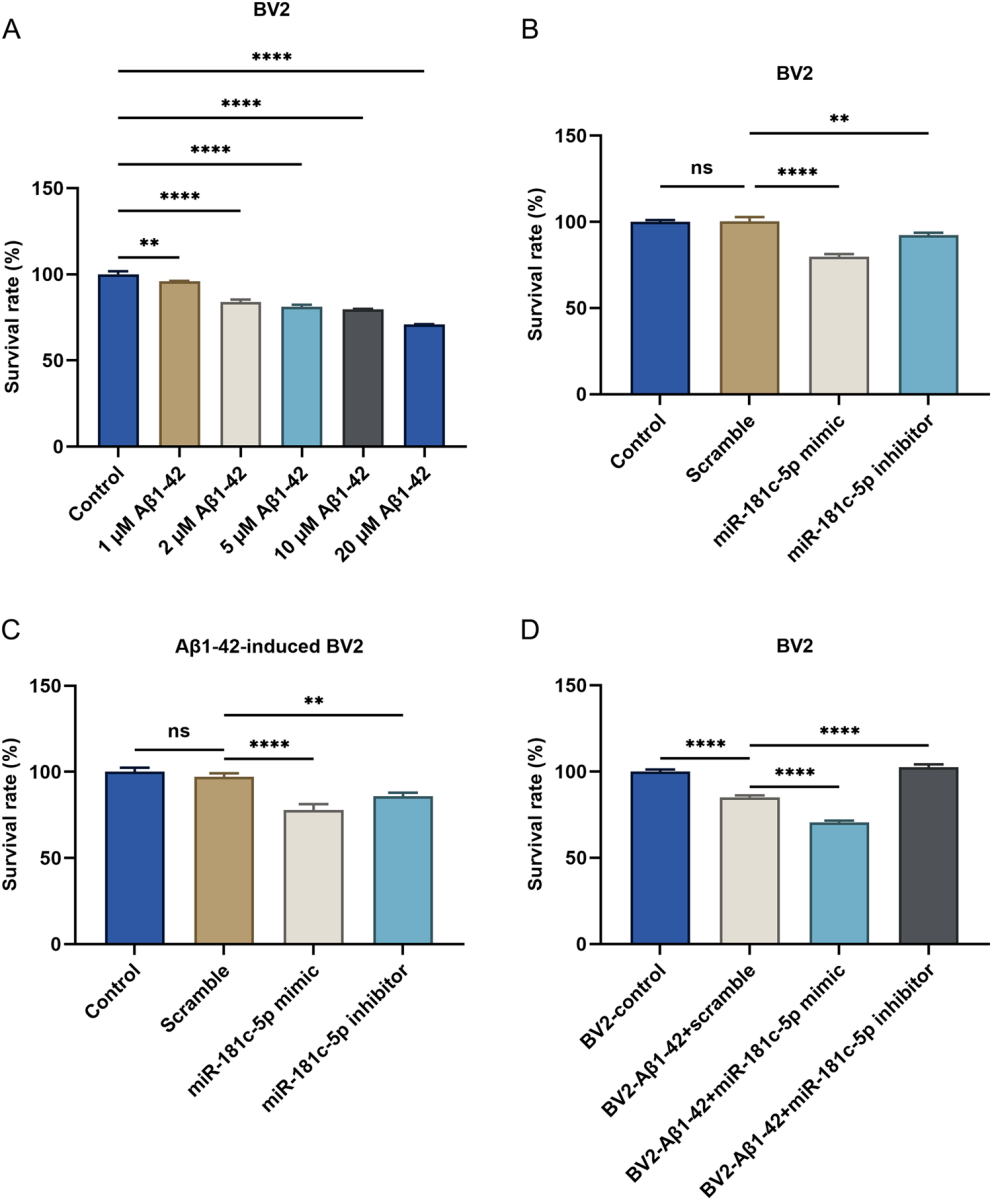
The supernatant of BV2 microglial cells with miR-181c-5p downregulation improves the survival of BV2 cells under Aβ1-42 stress. (**A**) CCK-8 of the survival of BV2 microglial cells administered with distinct doses of Aβ1-42. (**B**) Assessment of the survival of BV2 microglial cells with transfection of scramble sequence, miR-181c-5p mimic, or inhibitor. (**C**) The viability of 5 μM Aβ1-42-induced BV2 microglial cells transfected with scramble sequence, miR-181c-5p mimic, or inhibitor. (**D**) The viability of 5 μM Aβ1-42-induced BV2 microglial cells transfected with scramble sequence, miR-181c-5p mimic, or inhibitor that were cocultured with the supernatant of BV2 cells with transfection of scramble sequence, miR-181c-5p mimic, or inhibitor. **, *p* < 0.01; ****, *p* < 0.0001; ns, *p* > 0.05. Aβ, amyloid β; miR-181c-5p, microRNA-181c-5p; CCK-8, Cell Counting Kit-8.

CCK-8 was used to test the survival of BV2 microglial cells after exposure to distinct doses of Aβ1-42. Aβ1-42 notably lowered the survival of BV2 cells in a dose-independent manner (Fig. 3A). In BV2 cells, upregulated miR-181c-5p by its mimic decreased the survival of BV2 cells, with opposite effects under its inhibitor (Fig. 3B). Similar findings were observed under 5 μM Aβ1-42 stress (Fig. 3C). BV2 cells with 2 h transfection of scramble sequence, miR-181c-5p mimic, or inhibitor were cultivated by 5 μM Aβ1-42 for 6 h. Next, the supernatant from BV2 cells transfected with scramble sequence, miR-181c-5p mimic, or inhibitor was gathered and added to the above cells for 24 h cultivating. It was found that 5 μM Aβ1-42-induced BV cells had lower viability following coculturing with the supernatant of BV2 cells, which was further worsened by miR-181c-5p mimic, with opposite effects by its inhibitor (Fig. 3D). Hence, the supernatant of BV2 cells with miR-181c-5p downregulation potentially improved the survival of BV2 cells under Aβ1-42 stress.

### miR-181c-5p heightens apoptosis in Aβ1-42-induced BV2 microglial cells

**Figure 4:**
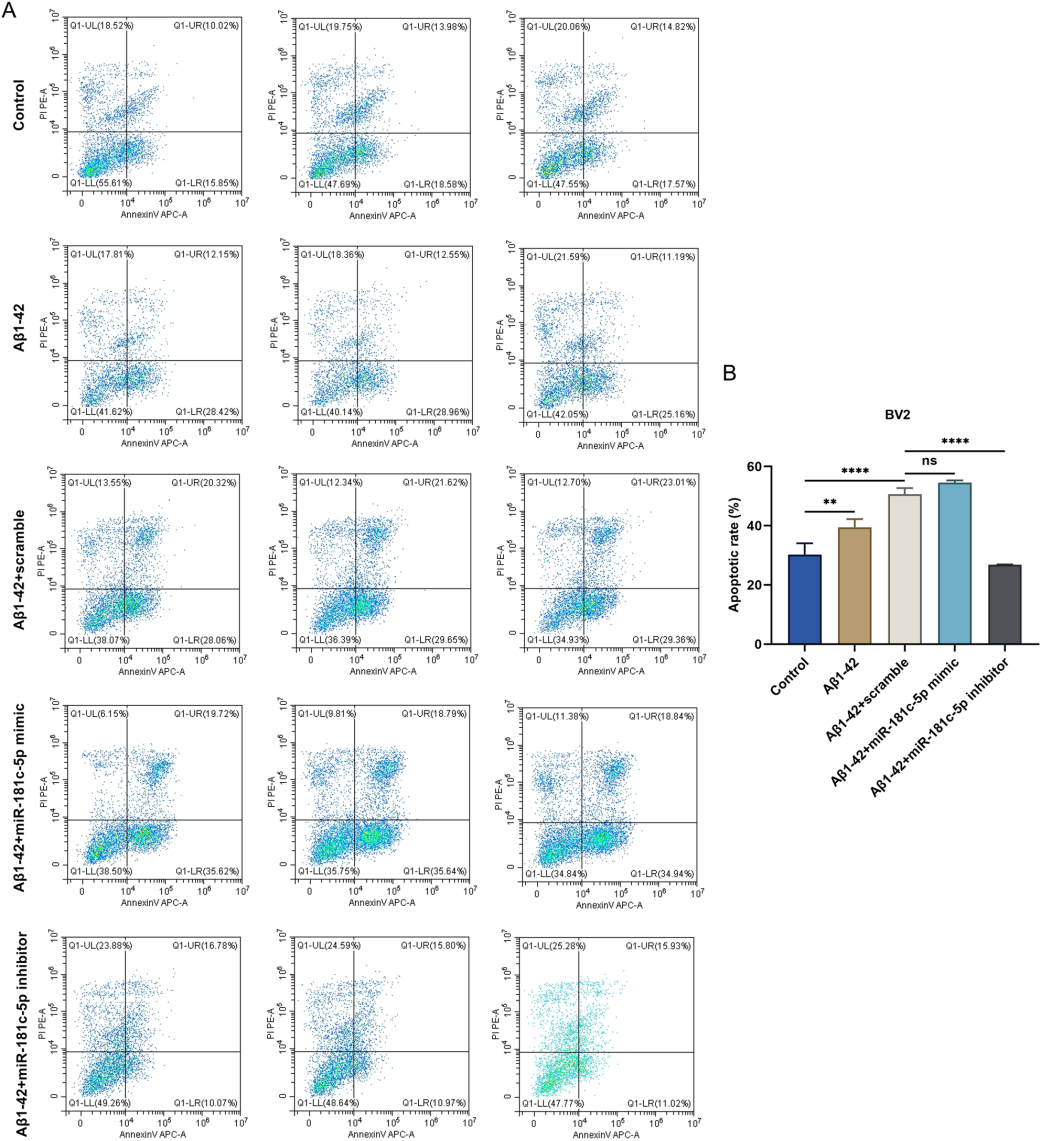
miR-181c-5p enhances the apoptotic level of Aβ1-42-induced BV2 microglial cells. (**A**, **B**) Annexin-V/PI staining of apoptosis in BV2 microglial cells with controls, 5 μM Aβ1-42 or/and scramble sequence, miR-181c-5p mimic, or inhibitor transfection. **, *p* < 0.01; ****, *p* < 0.0001; ns, *p* > 0.05. PI, propidium iodide; PE-A, phycoerythrin-area under the curve; UL, upper left; UR, upper right; LL, lower left; LR, lower right; APC-A, allophycocyanin-area under the curve; Aβ, amyloid β; miR-181c-5p, microRNA-181c-5p.

The impact of altered miR-181c-5p on BV2 microglial cell apoptosis was then measured using Annexin-V/PI staining. The 5 μM Aβ1-42-induced BV2 cells exhibited elevated apoptosis relative to controls (Fig. 4A-B). Notably, miR-181c-5p mimic modestly improved the Aβ1-42-induced apoptosis in BV2 cells. In addition, miR-181c-5p inhibitor notably attenuated apoptosis in 5 μM Aβ1-42-administered BV2 cells. Thus, miR-181c-5p may have improved the level of apoptosis in Aβ1-42-induced BV2 microglial cells.

### The downregulation of miR-181c-5p inhibited the phagocytosis of Aβ by Aβ1-42-induced BV2 microglial cells

**Figure 5:**
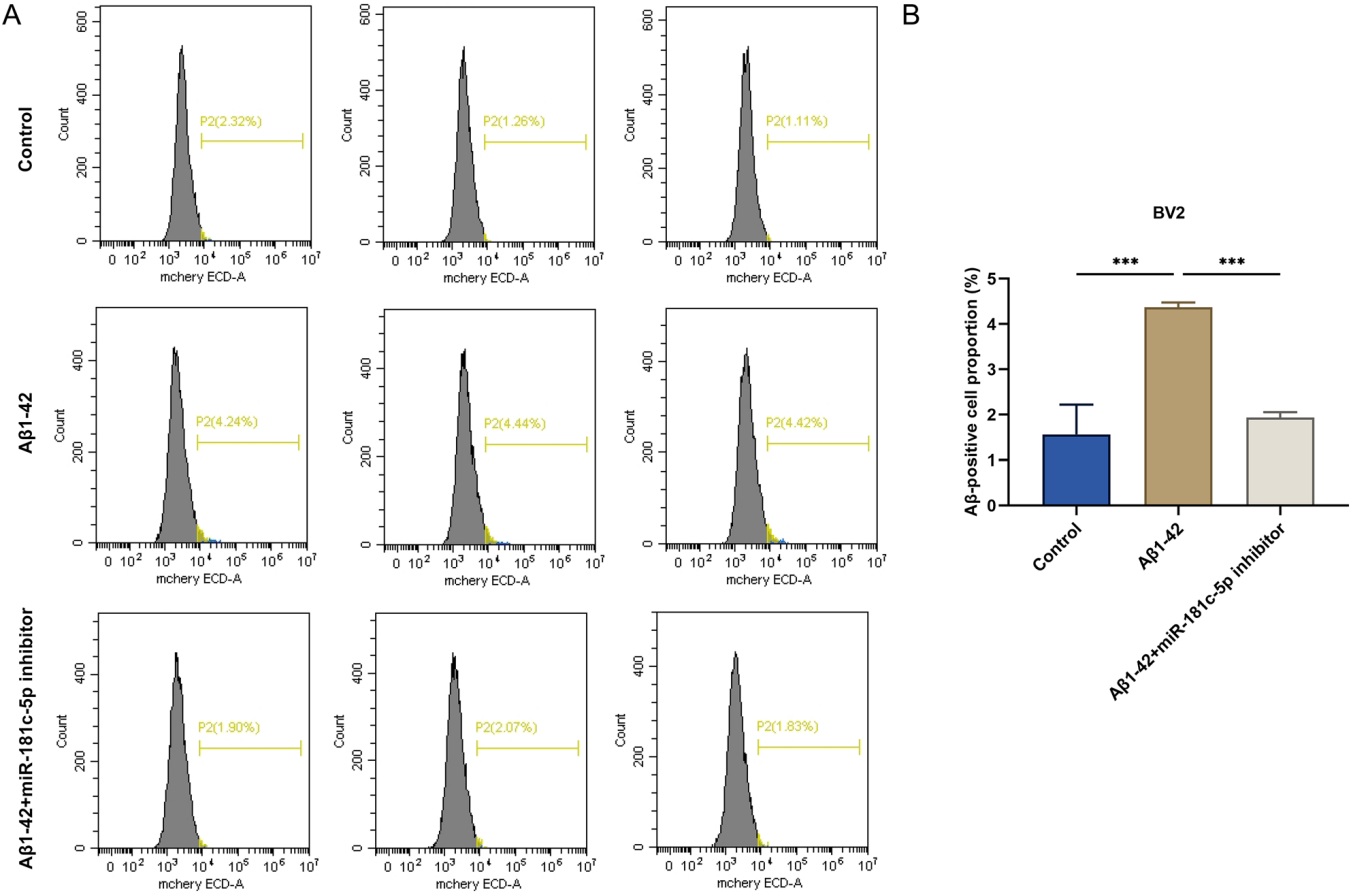
The downregulation of miR-181c-5p inhibited the phagocytosis of Aβ by Aβ1-42-induced BV2 microglial cells. (**A**, **B**) The Aβ-positive cell proportion in BV2 microglial cells with controls, 5 μM Aβ1-42 or/and miR-181c-5p inhibitor transfection by flow cytometric analysis. ***p < 0.001. Aβ, amyloid β; ECD-A, electron capture detector-A; miR-181c-5p, microRNA-181c-5p.

Flow cytometric analysis was used to measure the Aβ-positive cell proportion in BV2 microglial cells. Compared with controls, the 5 μM Aβ1-42-induced BV2 cells presented a higher Aβ-positive cell proportion (Fig. 5A-B). However, miR-181c-5p inhibitor lowered the Aβ-positive cell proportion in Aβ1-42-exposed BV2 cells. Altogether, lowering miR-181c-5p level potentially hindered Aβ phagocytosis of Aβ1-42-administered BV2 cells.

### miR-181c-5p downregulation facilitates the release of inflammatory cytokines in Aβ1-42-induced BV2 microglial cells

**Figure 6:**
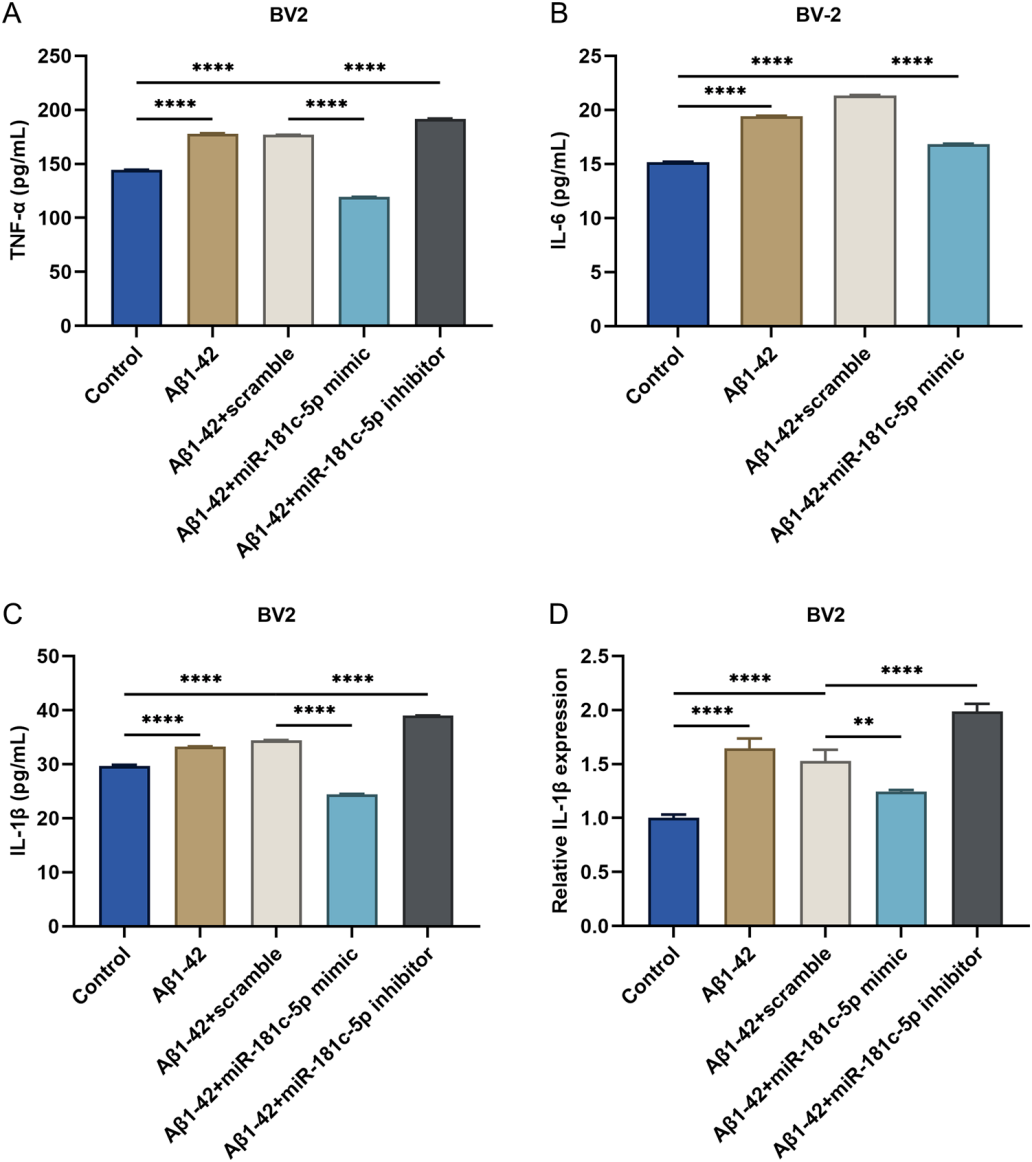
miR-181c-5p downregulation facilitates the release of inflammatory cytokines in Aβ1-42-induced BV2 microglial cells. (**A**–**C**) ELISA for measuring the levels of TNF-α, IL-6, and IL-1β in the supernatant of BV2 cells with controls, 5 μM Aβ1-42 or/and scramble sequence, miR-181c-5p mimic, or inhibitor transfection. (**D**) Transcript level of IL-1β in BV2 cells with controls, 5 μM Aβ1-42 or/and scramble sequence, miR-181c-5p mimic, or inhibitor transfection. **, *p* < 0.01; ****, *p* < 0.0001. TNF-α, tumor necrosis factor-α; Aβ, amyloid β; miR-181c-5p, microRNA-181c-5p; IL, interleukin; ELISA, enzyme-linked immunosorbent assay.

It was found that TNF-α, IL-6, and IL-1β presented the elevated level in the supernatant of BV2 microglia cells under 5 μM Aβ1-42 conditions (Fig. 6A–C). miR-181c-5p mimic markedly decreased their level in BV2 cells with Aβ1-42 administration. In contrast, miR-181c-5p inhibitor aggravated the Aβ1-42-induced increase of TNF-α, and IL-1β in BV2 cells. Afterwards, we measured the transcript level of IL-1β in BV2 microglial cells using RT-qPCR. IL-1β was elevated in 5 μM Aβ1-42-administered BV2 cells (Fig. 6D). Its level was reduced by miR-181c-5p mimic in Aβ1-42-induced BV2 cells. In contrast, miR-181c-5p inhibitor improved its level in BV2 cells under Aβ1-42 stress. As a consequence, miR-181c-5p downregulation facilitated the release of inflammatory cytokines in Aβ1-42-administered microglia.

### miR-181c-5p downregulation alleviates the phosphorylation of P38 in Aβ1-42-induced SH-SY5Y cells

**Figure 7:**
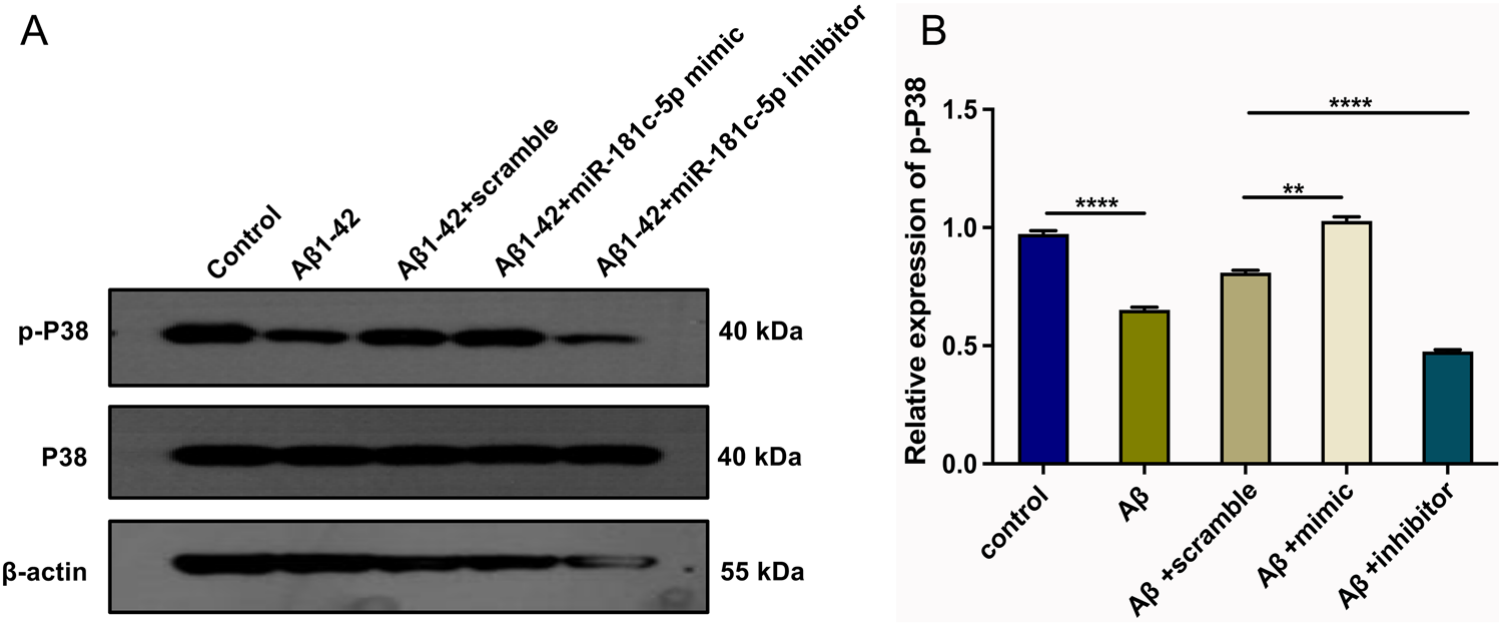
miR-181c-5p downregulation alleviates the phosphorylation of P38 in Aβ1-42-induced SH-SY5Y cells. (**A**-**B**) Western blot detecting the levels of p-P38 and P38 in SH-SY5Y cells with controls, 5 μM Aβ1-42 or/and scramble sequence, miR-181c-5p mimic, or inhibitor transfection. β-actin was utilized as a control. Aβ, amyloid β; miR-181c-5p, microRNA-181c-5p; p-P38, P38 phosphorylation.

Western blot was conducted to investigate the influence of miR-181c-5p on P38 activity in SH-SY5Y cells. It was shown that p-P38 level was notably downregulated in Aβ1-42-induced SH-SY5Y cells (Fig. 7A-B). miR-181c-5p mimic improved the level of p-P38 under Aβ1-42 stress. Conversely, its level was attenuated by miR-181c-5p inhibitor in Aβ1-42-induced SH-SY5Y cells. However, Aβ1-42 and miR-181c-5p did not influence P38 expression in SH-SY5Y cells. Altogether, downregulated miR-181c-5p mitigated the phosphorylation of P38 in Aβ1-42-induced SH-SY5Y cells.

## Discussion

Accumulated senile plaque in the brain is the dominating pathological feature of AD^10^. Aβ-42, which constitutes the major component of senile plaques, can induce various pathological alterations^24^, such as chronic inflammation, oxidative damage, nerve tangle formation, and progressive synaptic damage^25^. Although major efforts have been made to develop therapies^26^, little progress has been achieved, in part due to an incomplete understanding of the pathogenesis^27^. Aβ deposits possibly begin years or even decades before the onset of dementia symptoms^28^. Patients with late-onset AD in particular show impaired Aβ clearance instead of excessive Aβ generation^29^, so facilitating Aβ clearance represents a promising approach to treating AD^30^.

MicroRNAs (miRNAs) are a class of short, non-coding single-stranded RNAs, typically 22–24 nucleotides in length, that regulate gene expression post-transcriptionally by binding to messenger RNA (mRNA) molecules^15^. Abnormal expression of miRNAs has been demonstrated in AD^31–33^. For instance, miR-31 has been shown to improve cognitive abilities in Alzheimer’s patients and reduce Aβ accumulation^34^. Upregulation of miR-374b-5p in AD inhibits Aβ-induced neurotoxicity and neuroinflammation^35^. In AD, miR-30a-5p triggers Aβ generation by downregulating the non-amyloidogenic pathway^36^. Further investigation into the roles and mechanisms of miRNAs in AD may provide crucial insights into disease progression and the potential use of miRNAs as biomarkers for disease prevention^37^. Previous studies have reported reduced levels of serum miR-181c in AD patients, suggesting its potential as a minimally invasive biomarker for AD^38^. Additionally, decreased levels of miR-181c have been shown to be associated with elevated plasma levels of Aβ1-40 and brain vulnerability in normal aging^39^. Therefore, in this study, we aim to further investigate the impact of miR-181c-5p on Aβ accumulation.

Herein, we first performed normalization and differential analysis on the AD-related dataset GSE110226. We also constructed a PPI network for AD genes. Finally, through hypergeometric analysis, we identified the upstream regulatory genes miR-181c-5p. Subsequently, we conducted experiments on miR-181c-5p. Firstly, we established Aβ1-42-induced SH-SY5Y cell models. Consistent with serum level^40^, miR-181c-5p level was markedly downregulated in Aβ1-42-administered SH-SY5Y cells. Prior research has shown that serum miR-181c-5p is potentially associated with AD and depression^40^. Thus, aberrant miR-181c-5p expression might be causative in the initiation and progression of AD.

Microglia represent the sole form of resident macrophages within the central nervous system^41^. Under physiological conditions, microglia participate in maintaining brain homeostasis^42^. However, under abnormal stress, microglia are capable of quickly expanding the cell process, migrating to the injured sites, removing harmful substances, and inducing immune responses through the release of proinflammatory cytokines, especially TNF-α, IL-6, and IL-1β^43^. Activated microglia are capable of taking in soluble or fibrillar Aβ as well as degrading such peptides via proteasome signaling. Aberrantly increased soluble Aβ level correlates to elevated Aβ generation as well as lowered Aβ clearance^44^. Microglia, which have a high sensitivity to inflammatory stimuli, exhibit impaired phagocytic capacity as AD progresses^45^. The degradation of Aβ aggregates following phagocytosis is very slow, thus the effectiveness of microglia clearing away Aβ remains a key issue. Our results suggested that miR-181c-5p effectively regulated the activation of BV2 microglia under physiological and pathological (Aβ1-42) conditions. miR-181c-5p upregulation enhanced BV2 microglia phagocytosis of Aβ.

As AD progresses, microglial cells experience an age-related reduction in microglial capacity to interact with Aβ^46^. With aging, activated microglial cells ineffectively eliminate Aβ and the release of proinflammatory cytokines is elevated^47^. Evidence suggests that elevated proinflammatory cytokine levels exacerbate the degree of dementia in AD^48^. In addition, AD murine models exhibit a microglia-mediated neuroinflammation, and mitigating the inflammatory response of microglia represents an underlying approach for AD therapy^49,50^. miR-181c-5p downregulation may facilitate the release of inflammatory cytokines (TNF-α, IL-6, and IL-1β) in BV2 microglial cells under Aβ1-42 stress, indicating the inflammatory activation of microglia. Lipopolysaccharide induction has been found to downregulate the level of miR-181c-5p in microglia, and miR-181c-5p could lower TNF-α and IL-1β level as well as mitigate apoptosis^51^.

The phosphorylation of p38 was downregulated in SH-SY5Y cells under Aβ1-42 stress, which was further reduced by miR-181c-5p suppression, indicating that miR-181c-5p might participate in AD partly by modulating p38 phosphorylation. However, our findings will need to be further proven in murine models of AD. In addition, there are several limitations of this study, we did not consider the impact of miR-181c-5p on the survival effects in Aβ1-42-exposed SH-SY5Y cells. The main purpose of this study is to explore how the downregulation of miR-181c-5p inhibits microglial cell phagocytosis of Aβ. Through literature research, it is known that activated microglial cells can absorb soluble or fibrillar Aβ and degrade these peptides through the proteasome signaling pathway. Therefore, we mainly investigated the survival effects of miR-181c-5p in Aβ1-42-exposed BV2 cells. In the next step of our research, we will further investigate whether miR-181c-5p exhibits the same effects in other cell types. In this experiment, miR-181c-5p had an effect on P38 expression after SH-SY5Y damage induction, but not on MAPK1 expression after Aβ1-42 induction of BV2 damage. Both MAPK1 and P38 are mitogen-activated protein kinases. But we did not explore whether or how different mitogen-activated protein kinases affect neurons.

## Conclusions

Our work unveiled the unique property of miR-181c-5p in AD based on its effects in Aβ1-42-administered SH-SY5Y and BV2 cells. The level and function of miR-181c-5p were impaired following Aβ1-42 stress. Downregulation of miR-181c-5p can weaken the response of microglia to Aβ Phagocytosis exacerbates neuroinflammation. This work shed light on the essential role of miR-181c-5p in microglial function and its potential as a pharmacological target of AD. (Supplementary information).

### Supplementary Information

Supplementary Information.

## Data Availability

Sequence data that support the findings of this study was download from the public database online at the GEO database under accession numbers GSE110226.
