# Supplementary figures and images for: Downregulation of miR-181c-5p in Alzheimer’s disease weakens the response of microglia to Aβ phagocytosis
Source: Sci Rep. 2024 May 20;14:11487. doi: 10.1038/s41598-024-62347-x (PMC11106282; doi:10.1038/s41598-024-62347-x)

SH-SY5Y-Actin

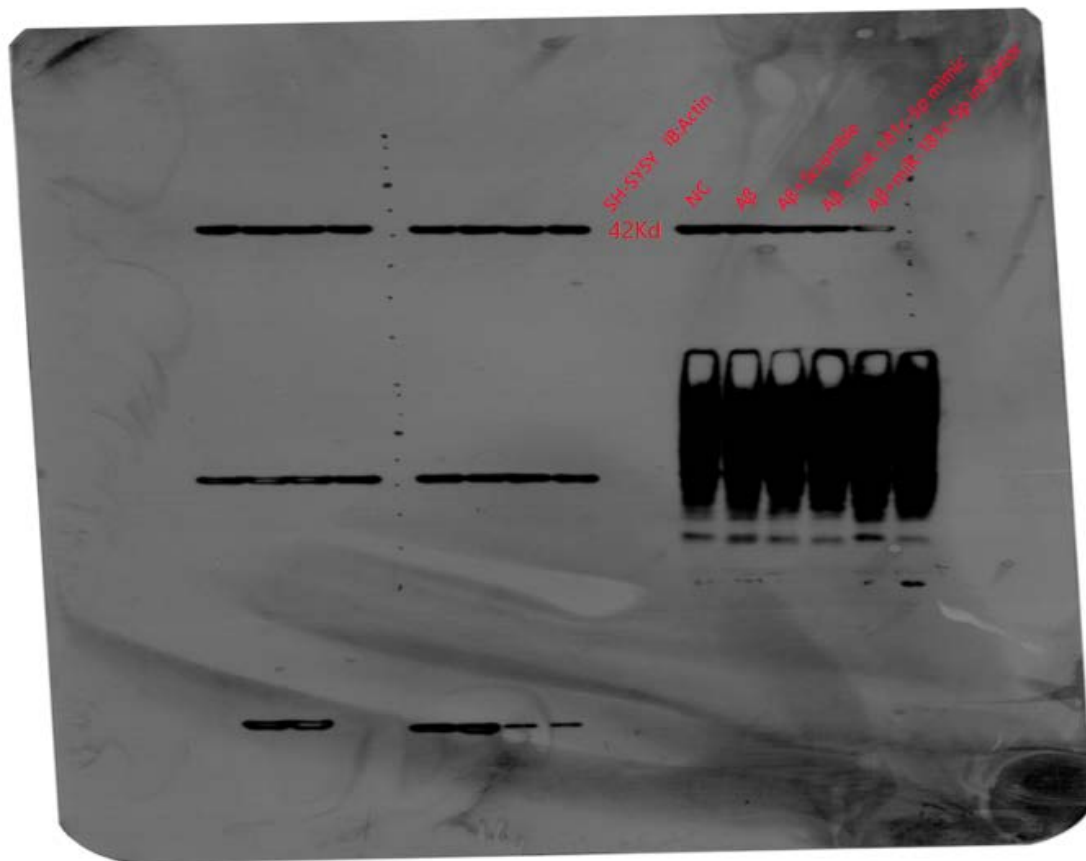

SH-SY5Y-P38-40Kd

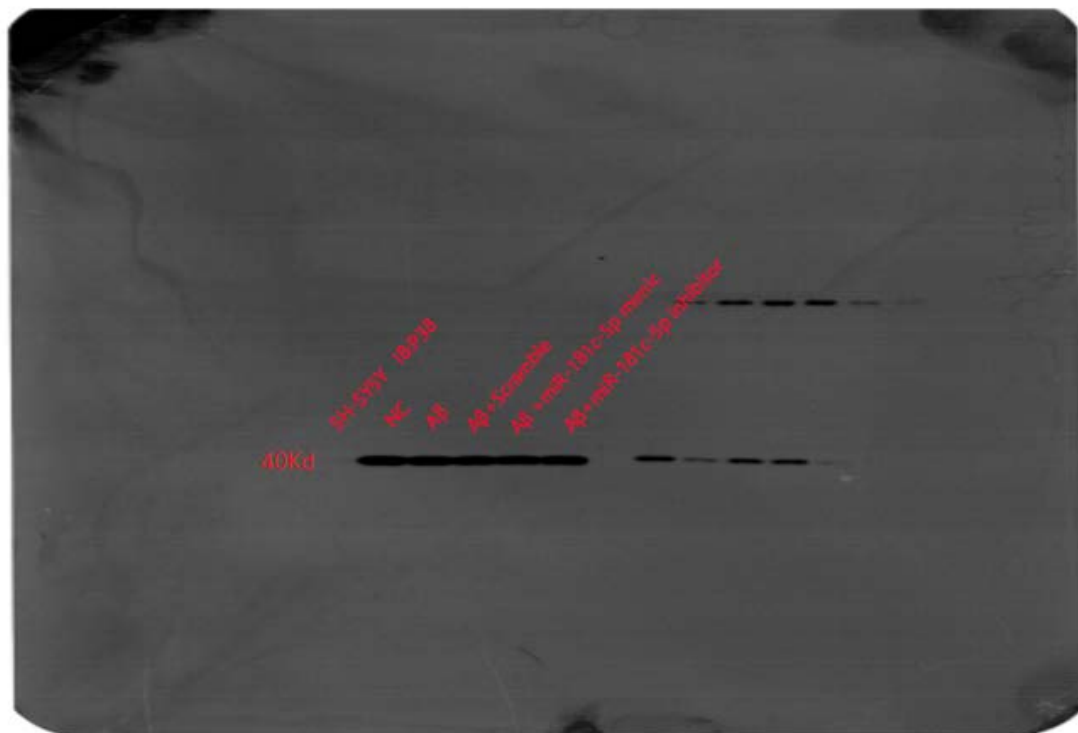

SH-SY5Y-p-P38-40Kd

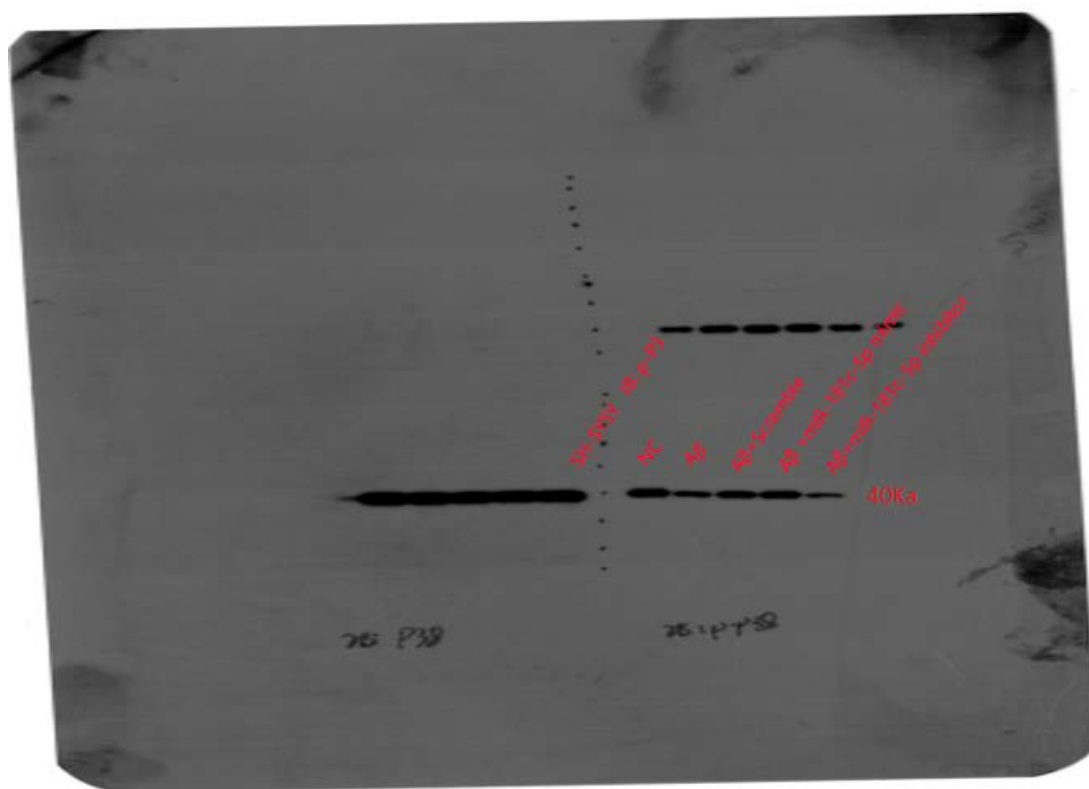

Supplement: Supplementary file 1 — Supplementary Information. [file 41598_2024_62347_MOESM1_ESM.pdf]
